# Surgical excision with left atrial reconstruction of a primary functioning retrocardiac paraganglioma

**DOI:** 10.1186/1749-8090-8-22

**Published:** 2013-01-29

**Authors:** María Teresa González López, Sergio González González, Esteban Sarria García, Stella González Romero, Julio Gutiérrez de Loma

**Affiliations:** 1 Cardiovascular Surgery Department, Carlos Haya Regional Hospital, Carlos Haya Avenue, s/n. 29010, Málaga, Spain; 2 Endocrinology Department, Carlos Haya Regional Hospital, Málaga, Spain

**Keywords:** Paraganglioma, Pheochromocytoma, Cardiac tumor, Left atrium, Cardiopulmonary bypass

## Abstract

About 2% of all paragangliomas are located in the chest, and a few have been described to be found in the heart. Primary cardiac paragangliomas are extremely uncommon tumors and surgical experience with this neoplasm is limited. Treatment strategies described in the literature have included simple excision, excision with reconstruction, autotransplantation after excision of the tumor and even orthotopic cardiac transplantation, depending on the extent of disease. A primary retrocardiac paraganglioma catecholamine-productive was identified in an asymptomatic 49–year old female associated to familial pheochromocytoma-paraganglioma syndrome caused by germline mutation of the gen which codifies for the subunit B of succinate dehydrogenase enzyme (SDHB). The neoplasm was surgically excised from the posterior surface of the left atrium via median sternotomy using cardiopulmonary bypass. Direct ligation of feeding vessels of the tumor along with left atrial reinforcement using a pericardial patch was performed. The post-operative course was uneventful, with normalization of catecholamine secretion and no recurrence at three-month follow-up. We review the current literature about this exceptional cardiac tumor, pathophysiological conditions and options for surgical management.

## Background

Pheochromocytomas are catecholamine-producing neuroendocrine tumors arise from the chromaffin cells of the embryonic neural crest. It is estimated that the annual incidence is approximately 0.8 per 100.000 persons and they occur most often in the fourth to fifth decade [1]. They are highly vascularised but usually benign tumors, although about 10% of pheochromocytomas are found to be malignant at the time of the primary tumor is discovered.

About 85–90% of them develop in the abdomen (they are usually found within one or both adrenal glands) and only 10% originates from extra-adrenal sites. Extra-adrenal pheochromocytomas (often described as extra-adrenal paragangliomas) originate in the ganglia of the sympathetic nervous system and most are located within the abdomen (celiac, superior mesenteric, inferior mesenteric ganglia and organ of Zuckerkandl), head and neck (3%, including carotid and vagal bodies), urinary bladder (1%) and mediastinal compartments (less than 1%) [2].

Thoracic paragangliomas are the most uncommon location and they can be divided into two groups: those located in the anterior mediastinum (arise from parasympathetic paraganglia) and located in the posterior mediastinum (sympathetic chain). Primary cardiac pheochromocytomas (paragangliomas) are extremely rare, occurring in only 0.001%–0.003%, with female predominance [3].

In general terms, the clinical manifestations of these tumors are varied, not particularly specific and it depends on the secretion of excessive amounts of catecholamines, such as noradrenaline (norepinephrine) and adrenaline (epinephrine). Although these tumors may be a cause of hypertension, they are the underlying cause of only about 0.01% of cases of high blood pressure.

About 75% of pheochromocytomas are sporadic and the remaining 25% are hereditary. Mutations of the genes which codify for the subunits D, A, C y B of succinate dehydrogenase enzyme (SDHD, SDHA, SDHC and SDHB) involved in the Krebs cycle can lead to tumorogenesis in chromaffin cells and they have been identified as causing familial adrenal pheochromocytoma and extra-adrenal paraganglioma; moreover, these tumors may also occur in the multiple endocrine neoplasia (MEN) syndrome type 2A and 2B, von Hippel-Lindau disease or von Recklinghausen’s neurofibromatosis [4].

The diagnosis of these tumors can be established by measuring catecholamines and metanephrines in plasma or 24-hour urine along with imaging techniques such as computed tomography (CT), magnetic resonance imaging, iodine-123-marked metaiodobenzylguanidine (MIBG) scan and positron emission tomography (PET) scan [5].

Treatment of pheochromocytomas involves blocking the effect of catecholamines with an alpha adrenergic blocker and after, it then becomes safe to surgically remove the tumor. Although surgical resection is the treatment of first choice in most part of cases, extra-adrenal localizations (such as those paragangliomas originated in cardiac structures) involve an uncommon and complex surgical approach for a complete removal.

We describe a case of surgical excision of a primary functioning retrocardiac paraganglioma associated with hereditary syndrome and we review the relevant literature about the peculiar features and surgical strategies for removal of this cardiac neoplasm.

## Case presentation

We report a case of an asymptomatic 49-year old Caucasian female, with hereditary type 4 pheochromocytoma-paraganglioma syndrome caused by germline mutation (alanine–43–proline) of the gen which codifies for the subunit B of succinate dehydrogenase enzyme (SDHB) of the mitochondrial complex II. She had a family history of extra-adrenal paraganglioma: genetic testing had revealed the gen SDHB mutation for eight first- and second-degree relatives and carotid body paraganglioma had been resected in three of them. Urinary and plasma levels of catecholamines (noradrenaline and adrenaline) and their metabolites (normetanephrine) were elevated and computed tomography of the abdomen ruled out adrenal gland involvement.

**Figure 1:**
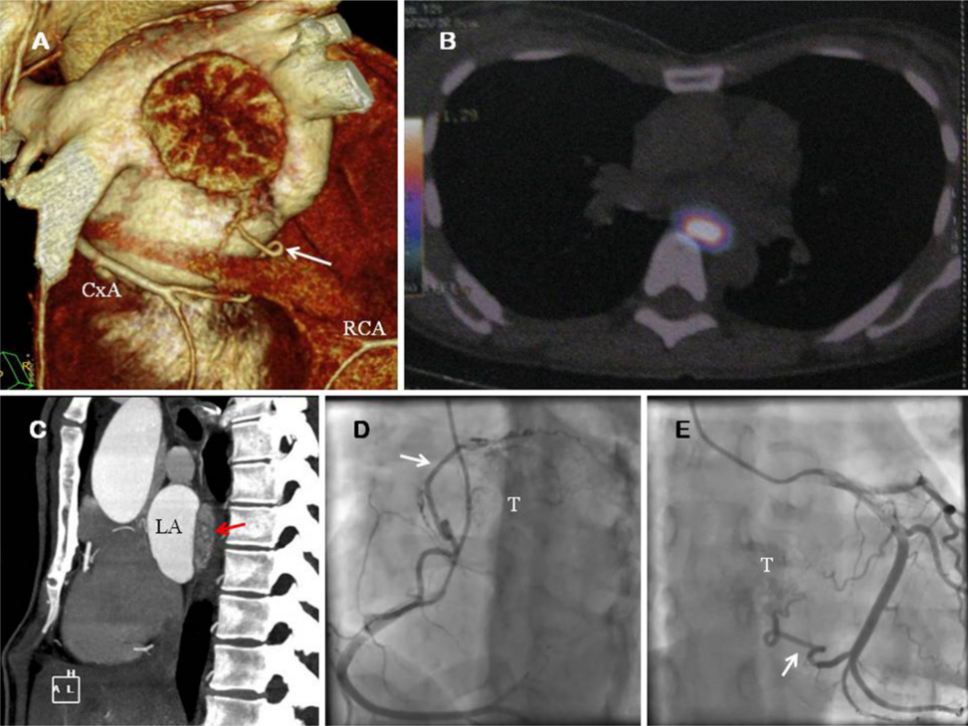
**A. Computed tomography imaging reconstruction of the heart.** The tumor is showed. The collateral vessel (white arrow) from the circumflex artery (CxA) is observed. **B.** 18-F DOPA PET showed a 16 × 25 × 36 mm extracardiac mass protruding within the LA. Presence of metabolic activity was detected. **C.** Computed tomography (axial view) showed the presence of the large mass (red arrow). **D.** Coronary angiography. The coronary blood supply of the superior aspect of the paraganglioma (T) emerged from a large collateral vessel (white arrow) from the right coronary artery (RCA). **E.** Coronary angiography. Proximal and distal collaterals from CxA were the feeding vessels (white arrow) of the inferior aspect of the tumor (T).

Total body MIBG scintigraphy scan and ^18^F-dihydroxy-phenyl-alanine (^18^F-DOPA) PET scan showed a primary retrocardiac mass with metabolic activity. Metastasic disease was not found and no other intrathoracic masses were identified. Transesophageal echocardiography (TOE) and TC imaging of the heart showed the large mass (40 × 20 mm), extrinsic to the heart, on the posterior wall of the left atrium (LA) (Figure 1A-C). Coronary angiography and aortography were performed: the tumor received dual blood supply from both right and left coronary arteries (Figure 1D-E). No feeding vessels from the aortic arch or thoracic descending aorta (bronchial arteries) were demonstrated.

**Figure 2:**
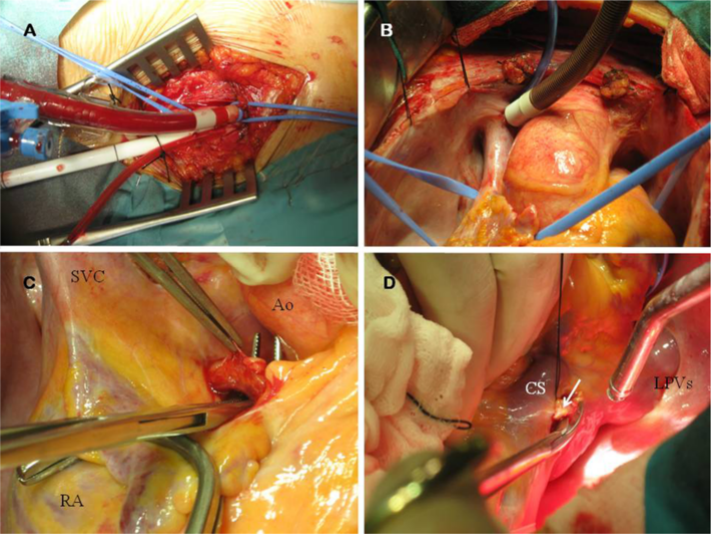
**A. Peripheral perfusion through right femoral artery and vein. B.** Superior vein cavae (SVC) drainage through Pacifico cannula.**C.** The large collateral vessel from the RCA was dissected and ligated. *RA*: *Right atrium*. *Ao*: *Aorta*. **D.** The two collateral vessels from CxA (white arrow) were also ligated for an optimal control of bleeding during the excision of the tumor. *CS*: *Coronary sinus*. *LPVs*: *Left pulmonary veins*.

Alpha-adrenergic pharmacologic therapy (phenoxybenzamine) was instituted before operation for the catecholamine-secreting tumor. She underwent surgical removal of the retrocardiac tumor via median sternotomy. As part of our initial surgical planning, cardiopulmonary bypass (CPB) was started through right femoral artery and venous cannulation (right femoral vein and superior vein cava) (Figure 2A-B) because of surgical excision could be technically difficult by the position of the tumor. Moderate hypothermia (31°C) was instaured and the heart was arrested using antegrade cold blood cardioplegia. The tumor was found as an “imprisoned” mass between the four pulmonary veins: it extended from the right to the left superior pulmonary vein, and caudally, between the inferior pulmonary veins. The lesion was confined to this retrocardiac area as a firm adherent mass, although it was not adherent to adjacent structures.

Direct ligation of feeding vessels was performed for control of bleeding: the collaterals vessels from the right coronary artery (RCA) and circumflex artery (CxA) were ligated (Figure 2C-D). TOE was used to confirm successful interruption of blood flow to the tumor and no electrical changes were noted.

**Figure 3:**
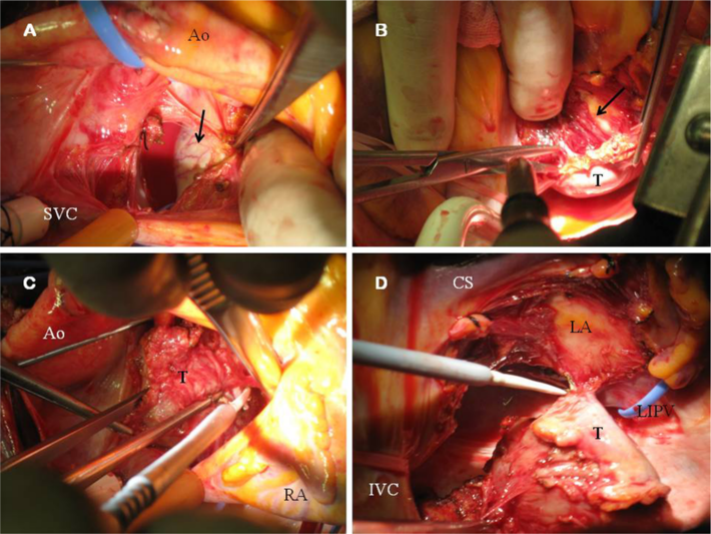
**A. Surgeon’s view.** Superior aspect of the tumor (black arrow) is showed. Neovascularization of the smooth surface is observed. **B.** Endocardium of the LA was exposed (black arrow) during surgical excision (suspended heart). **C.** Surgeon’s view. Dissection of the superior aspect of the tumor (T). **D.** Paraganglioma (T) after totally excision from the LA. *IVC*: *Inferior vein cava*. *LIPV*: *Left inferior vein cava*.

**Figure 4:**
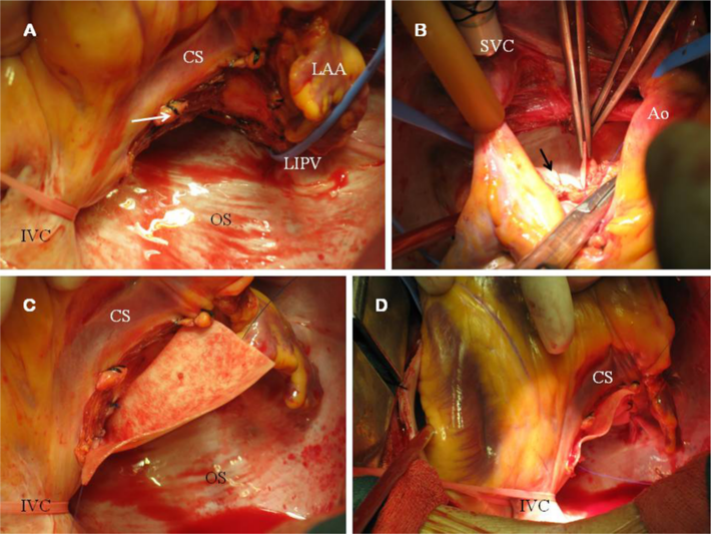
**A. LA after removal of the tumor.** The operative photograph shows the distal collateral vessel (white arrow) from CxA (after ligation). Endocardium of the LA remained in situ. *LAA*: *Left atrial appendage*. *OS*: *Oblique sinus*. **B.** Bovine pericardial patch (black arrow) was used for left atrial reconstruction. The suture was started from the left atrial roof. **C-D.** Pericardial patch was placed on the posterior wall of the LA using a polypropylene running suture.

**Figure 5:**
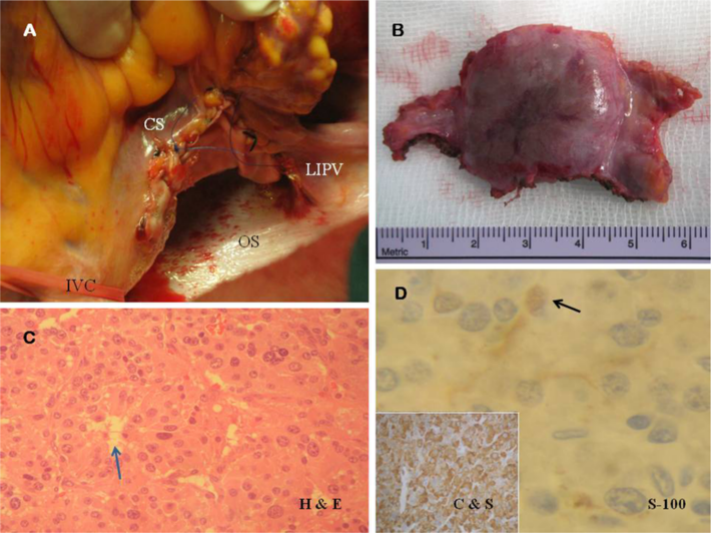
**A. Posterior LA after pericardial replacement. B.** Macroscopic examination (6 × 3 × 1.5 cm fragment). A well-demarcated homogeneous, smooth surfaced and highly vascularized tumor (2.8 × 2.5 × 1.5 cm) was found, with clear surgical margins. **C.** Histological examination. Haematoxylin and eosin staining (H & E, 40x magnification) showing the “zellballen” packeting of cells, without atypia, surrounded by capillary network (blue arrow). **D.** Immunohistochemical analysis (40x magnification) revealed the specific neuroendocrine markers (chromogranin and synaptophysin, C & S). S-100 protein staining technique (100x magnification) revealed the sustentacular cell network (black arrow).

Following excision of the superior pericardial reflection (Figure 3A), the left posterior atrial wall was exposed and the superior aspect of the tumor was excised. Then, the heart was suspended for removal of the inferior aspect of the tumor. There was no a plane of cleavage between the LA and the tumor, and atrial myocardium was also removed (Figure 3B-C). The division of great vessels and LA was not necessary to access the tumor and it was completely excised en bloc (Figure 3D). Coronary sinus, pulmonary veins and left atrial appendage were not involved. Adequate disease-free margins were achieved and the denuded LA was reconstructed using a bovine pericardial patch (Figures 4A-D and 5A) and it was reinforced with surgical sealant.

CPB time was 143 minutes and aortic cross-clamping time was 105 minutes. Intraoperative TOE revealed a complete excision of the tumor and no perioperative hypertensive crisis on palpation of the tumor were demonstrated. The patient was then weaned from bypass successfully.

Histological examination confirmed the paraganglioma and malignant changes were not found (Figure 5B-C). Chromogranin and synaptophysin were positive at immunohistochemistry, and sustentacular cells were positive for S–100 protein staining (Figure 5D).

The post-operative course was uneventful, no bleeding was detected, sinus rhythm was maintained and antihypertensive therapy was not necessary. She was discharged at 8^th^ post-operative day and short-term anticoagulation was started for prevention of LA thrombus formation due to transient endothelial dysfunction. At 3–month follow-up, she remains asymptomatic and biochemical evaluations have confirmed the normalization of levels of catecholamines.

## Discussion

Cardiac paraganglionic tumors are rare neoplasms derived from the neural crest in the mediastinum, associated with the autonomic nervous system, which account for 0.3% of all mediastinal neoplasms and less than 1% of all primary cardiac tumors [6]. They arise from either the branchiomeric paraganglia (coronary or aortopulmonary) or the visceral-autonomic paraganglia (atrium or interatrial septum) and less than 50% are functional; the majority of functional tumors are intraadrenal pheochromocytomas, being uncommon at the heart.

Edwin Besterman (St. Mary’s Hospital, London) was the first author to report a successful resection on CPB of a cardiac pheochromocytoma in the LA in 1974 [7] and to date, fewer than 50 cases have been reported in the medical literature [8,9] and they are typically located adjacent to or involving the LA [10], although these tumors have been described to be found in the interatrial septum [11], right atrium, inferior vein cava [12], root of the great arteries [13] or proximal part of coronary arteries [14], possibly explained by close proximity of paraganglionic cell nest.

Clinical presentation depends on the functional status of the paraganglioma and the location at the heart: paroxysmal hypertension due to secretion of catecholamines, chest pain [15], symptoms secondary to pericardial involvement or invasion of the conduction system [16] or inespecified symptoms such as fever or malaise. In cases of extension to valves, other primary cardiac tumors such as mixoma or angiosarcoma must be excluded. More unusual presentations have been reported such as acute myocardial infarction and stroke [17], although in some cases, the tumor can be an incidental finding. A curious feature of the tumor in our patient was the absence of symptoms despite to be a functioning paraganglioma.

In a short number of patients, cardiac paragangliomas can be associated to hereditary syndromes such as the Carney triad (pulmonary chondromas, gastric stromal tumors and extra-adrenal paragangliomas) [18] or familial pheochromocytoma-paraganglioma syndromes. In the reported case, the familial pheochromocytoma-paraganglioma syndrome type 4 had been previously detected. These syndromes involve a group of rare autosomal dominant disorders affecting sympathetic and parasympathetic paraganglia, caused by germline mutations of the genes SDHB, SDHC and SDHD, although some of these mutations can also be detected for patients with apparently sporadic pheochromocytomas [19].

Most cardiac paragangliomas are benign, but local invasion can occur and metastases have been reported in isolated cases [20,21]. Location in the heart is not particularly associated with familial syndromes, although malignant predisposition is increased with the SDHB mutation [22]. Because of this reason, long-term follow-up is mandatory in our patient due to the implications of this hereditary disorder. However, malignant primary paragangliomas of the heart are exceptional and to date, there are no reported cases associated to this mutation.

The preoperative goals for this type of tumors are to normalize hemodynamic variables such as blood pressure and avoid surges in catecholamine release with tumor manipulation during surgery. Nevertheless, preoperative alpha-1-adrenoceptor antagonist seems to have no benefit in maintaining intraoperative hemodynamic stability in patients with normotensive adrenal pheochromocytoma [23]. Although our patient showed normal preoperative levels of blood pressure despite to be a catecholamine-secreting tumor and it is important to bear in mind these recent considerations, no evidence about normotensive cardiac paragangliomas has been reported and we consider the alpha-blockade preparation for this isolated case.

Less than 40 cases about surgical excision of a cardiac paraganglioma have been described in adults [24] and the surgical treatment is similar to that of all other cardiac tumors; the approach can be either a median sternotomy or posterolateral thoracotomy depending on the location in the mediastium, but some technical details must be made.

Because they are highly vascular, some groups advocate the embolization before surgery to reduce perioperative bleeding [25] when surgical excision of a bulky tumor at a difficult location is planned. Although preoperative embolization has been widely described in the treatment of paragangliomas of the neck and carotid body, it has been reported in a few cases of mediastinal paragangliomas. For these situations, superselective angiography and subsequent embolization must be carried out 1 to 6 days preoperatively for a complete disappearance of the tumor blush and a safe surgical excision.

Nevertheless, in our patient, for prevention of a catastrophic haemorrhage during the time of the operation, a direct ligation of feeding vessels of the tumor was performed. To date, isolated cases of direct ligation at surgery have been described [26] and no comparative results between the two options are available.

As these tumors are of locally invasive nature and they have a less well-defined capsule than adrenal pheochromocytomas, a complete resection can be technically difficult by the position of the tumor and should be performed on CPB and cardioplegic arrest in the most part of cases in order to achieve disease-free excision margins. When the interatrial septum is involved, the anterior aspect of the septum must be preserved to avoid heart block; in other situations, the tumor invades surrounding vascular structures and resection may be extremely difficult. For selected patients, extensive cardiac reconstructive surgery may therefore be required, with its associated risks. Our patient underwent a successful complete resection of the “caged” tumor between the pulmonary veins along with reconstruction of the resected site and no complications were detected after surgery.

Reconstruction of the LA using either autologous or bovine pericardial patch has been previously described and, when a portion of the pulmonary veins is also infiltrated and excised, it must be repaired and reconstructed with the patch [27], although these cases are at risk for development of pulmonary stenosis at follow-up. Reconstruction of right ventricular outflow tract and pulmonary valve has also been reported when local invasion is detected [28] following the removal of the tumor en bloc with adjacent structures such as thymus, pericardium, part of pulmonary trunk and anterior leaflet of pulmonary valve.

Although surgical resection and reconstruction is the gold standard treatment for these tumors, this approach can be impossible due to extensive involvement of the great vessels or other structures of the heart. When total surgical resection is extremely difficult, cardiac autotransplantation has been described and, in selected cases, when a complete removal is not possible, orthotopic cardiac transplantation is a feasible technique in those paragangliomas that have direct coronary artery involvement, extend into the left ventricle or invade the atrioventricular groove [29]. In general terms, although operative mortality and overall survival seems favorable in series of patients with primary cardiac tumors and benefits of this technique include improved accessibility and ability to perform a complete tumor resection, there are few reports of transplantation for excision of cardiac paragangliomas in the literature and late results are unknown [29].

In the only reported case series including 14 patients over the last 30 years, at Mayo Clinic [30], Brown et al. have reported a safe resection of these tumors often under CPB with modest surgical risk, with one intraoperative death due to massive bleeding (in the only tumor of this serie involving the LA) and a total number of 10 patients were alive at follow-up (median follow-up: 2.3 years). The SDHB mutation was detected in two patients of the serie.

When a complete removal of the tumor is achieved, long-term prognosis seems to be excellent and survival of up to 14 years has been reported in a patient with excised mediastinal paraganglioma [31]. To date, surveillance can be done by following serum catecholamine levels, repeating CT scans and/or TOE, and most recently MIBG scan and PET imaging. Recent data support the superiority of 18F-DOPA PET-CT over 123–I–MIBG scintigraphy to assess disease extension in patients with recurrent paraganglioma [32] and it might be the preferred test in this patient follow-up because of the implications of the hereditary syndrome. However, there is no consensus on the treatment if the tumor does recur at follow-up.

Our case is relevant in that there were no classic signs of catecholamine excess despite to be a functioning tumor and it illustrates successful management of a benign left atrial primary paraganglioma associated with SDHB mutation, including direct ligation of feeding vessels of the tumor, complete excision and subsequent reinforcement of the LA, without evidence of recurrence at short-term follow-up.

## Conclusions

– Primary cardiac paragangliomas are exceptional tumors and surgical resection is the gold standard treatment.

– Control of feeding vessels, complete resection of the tumor and reconstruction of the resected sites are the goals for surgical management.

– Although treatment strategies can be varied according to the extent of the disease, in general terms, cardiac paragangliomas can be safely resected using well-established surgical techniques with appropriate surgical planning.

## Consent

Written informed consent was obtained from the patient for publication of this Case report and any accompanying images. A copy of the written consent is available for review by the Editor-in-Chief of this journal.

## Abbreviations

SDHB: Subunit B of succinate dehydrogenase enzyme;SDHD: Subunit D of succinate dehydrogenase enzyme;SDHA: Subunit A of succinate dehydrogenase enzyme;SDHC: Subunit C of succinate dehydrogenase enzyme;MEN: Multiple endocrine neoplasia syndrome;CT: Computed tomography;MIBG – scan: Iodine-123-marked metaiodobenzylguanidine – scan;PET – scan: Positron emission tomography – scan;(18F-DOPA) PET scan: ^18^F-dihydroxy-phenyl-alanine positron emission tomography scan;TOE: Transesophageal echocardiography;LA: Left atrium;CPB: Cardiopulmonary bypass;RCA: Right coronary artery;CxA: Circumflex artery;SVC: Superior vein cavae;RA: Right atrium;Ao: Aorta;CS: Coronary sinus;LPVs: Left pulmonary veins;IVC: Inferior vein cava;LIPV: Left inferior vein cava;LAA: Left atrial appendage;OS: Oblique sinus;H & E: Haematoxylin and eosin;C & S: Chromogranin and synaptophysin

## Competing interests

The authors declare that they have no competing interests.

## Authors’ contributions

MTGL: Design, data collection, photographs and writing article. SGG; ESG; SGR; and JGL: Critical revision and helped to draft the manuscript. All authors read and approved the final manuscript.

## References

[B1] Walther MM Keiser HR Linehan WM Pheochromocytoma: evaluation, diagnosis, and treatment World J Urol 1999 17 35 39 10.1007/s003450050102 10096149

[B2] Manger WM In search of pheochromocytomas J Clin Endocrinol Metab 2003 88 4080 4082 10.1210/jc.2003-031234 12970266

[B3] Pacheco N Marcos G Garcipérez FJ Pérez C Intrapericardial paraganglioma Rev Esp Cardiol 2010 63 116 117 10.1016/s1885-5857(10)70020-7 20089237

[B4] Elder EE Elder G Larsson C Pheochromocytoma and functional paraganglioma syndrome: no longer the 10% tumor J Surg Oncol 2005 89 193 201 10.1002/jso.20177 15719371

[B5] Tomasian A Lai C Ruehm S Krishnam MS Cardiovascular magnetic resonance and PET-CT of left atrial paraganglioma J Cardiovasc Magn Reson 2010 12 1 10.1186/1532-429X-12-1 20047692 PMC2817869

[B6] Huo JL Choi JC DeLuna A Lee D Fleischmann D Berry GJ Cardiac paraganglioma: diagnostic and surgical challenges J Card Surg 2012 27 178 182 10.1111/j.1540-8191.2011.01378.x 22273468

[B7] Besterman E Bromley LL Peart WS An intrapericardial pheochromocytoma Br Heart J 1974 36 318 10.1136/hrt.36.3.318 4824541 PMC458837

[B8] Avarot DJ Banner NR Cantor AM Location, localization, and surgical treatment of cardiac pheochromocytoma Am J Cardiol 1992 69 283 285 10.1016/0002-9149(92)91324-W 1731477

[B9] Jebara VA Uva MS Carpentier A Cardiac pheochromocytomas Ann Thorac Surg 1992 53 356 361 10.1016/0003-4975(92)91354-C 1731689

[B10] Tekin UN Khan IA Singh N Nair VM Vasavada BC Sacchi TJ A left atrial paraganglioma patient presenting with compressive dysphagia Can J Cardiol 2000 16 383 385 10744802

[B11] Cane ME Berrizbeitia LD Yang SS Mahapatro D McGrath LB Paraganglioma of the interatrial septum Ann Thorac Surg 1996 61 1845 1847 10.1016/0003-4975(96)00066-5 8651806

[B12] Rotker J Oberpenning F Scheld HH Hertle L Knichwitz G Hamm D Pheochromocytomas with extension into central vascular structures Ann Thorac Surg 1996 61 222 224 10.1016/0003-4975(95)00774-1 8561564

[B13] Otake Y Aoki M Imamura N Ishikawa M Hashimoto K Fujiyama R Aortico-pulmonary paraganglioma: case report and japanese review Jpn J Thorac Cardiovasc Surg 2006 54 212 216 16764311 10.1007/BF02670315

[B14] Birkenfeld A Bergmann M Bräsen JH Luft FC Neumann H A paraganglioma parasitizing the left circumflex coronary artery Am J Med 2004 116 787 788 10.1016/j.amjmed.2004.01.012 15144924

[B15] Khalid TJ Zuberi O Zuberi L Khalid I A rare case of cardiac paraganglioma presenting as anginal pain: case report Cases Journal 2009 2 72 10.1186/1757-1626-2-72 19159442 PMC2635122

[B16] Kennelly R Aziz R Toner M Young V Right atrial paraganglioma: an unusual primary cardiac tumor Eur J Cardiothorac Surg 2008 33 1150 1152 10.1016/j.ejcts.2008.02.031 18406162

[B17] Hayek ER Hughes MM Speakman ED Miller HJ Stocker PJ Cardiac paraganglioma presenting with acute myocardial infarction and stroke Ann Thorac Surg 2007 83 1882 1884 10.1016/j.athoracsur.2006.12.023 17462425

[B18] Colwell AS D’Cunha J Maddaus MA Carney’s triad paragangliomas J Thorac Cardiovasc Surg 2001 121 1011 1102 10.1067/mtc.2001.112820 11326257

[B19] Neumann HP Bausch B McWhinney SR Bender BU Gimm O Franke G Germ-line mutations in nonsyndromic pheochromocytoma N Engl J Med 2002 346 1459 1466 10.1056/NEJMoa020152 12000816

[B20] Chan KM Pontefract D Andrews R Naik SK Paraganglioma of the left atrium J Thorac Cardiovasc Surg 2001 122 1032 1033 10.1067/mtc.2001.115928 11689815

[B21] Arai A Naruse M Naruse K Tanabe A Yoshimoto T Iwama T Cardiac malignant pheochromocytoma with bone metastases Intern Med 1998 37 940 944 10.2169/internalmedicine.37.940 9868956

[B22] Neumann HP Pawlu C Peczkowska M Bausch B McWhinney SR Muresan M Distinct clinical features of paraganglioma syndromes associated with SDHB and SDHD gene mutations JAMA 2004 292 943 951 10.1001/jama.292.8.943 15328326

[B23] Shao Y Chen R Shen ZJ Teng Y Huang P Rui WB Preoperative alpha blockade for normotensive pheochromocytoma; is it necessary? J Hypertens 2011 29 2429 2432 10.1097/HJH.0b013e32834d24d9 22025238

[B24] Hamilton BH Francis IR Gross BH Intrapericardial paragangliomas (pheochromocytomas): imaging features Am J Roentgenol 1997 168 109 113 10.2214/ajr.168.1.8976931 8976931

[B25] Rakovich G Ferraro P Therasse E Duranceau A Preoperative embolization in the management of a mediastinal paraganglioma Ann Thorac Surg 2001 72 601 603 10.1016/S0003-4975(00)02293-1 11515906

[B26] Turley AJ Hunter S Stewart MJ A cardiac paraganglioma presenting with atypical chest pain Eur J Cardiothorac Surg 2005 28 352 354 10.1016/j.ejcts.2005.04.038 15990328

[B27] Ceresa F Sansone F Rinaldi M Patane F Left atrial paraganglioma: diagnosis and surgical managenement Interact Cardiovasc Thorac Surg 2010 10 1047 1048 10.1510/icvts.2009.231043 20197349

[B28] Lupinski RW Shankar S Agasthian T Lim CH Mancer K Primary cardiac paraganglioma Ann Thorac Surg 2004 78 43 44 10.1016/j.athoracsur.2004.01.015 15337082

[B29] Jeevanandam V Oz MC Shapiro B Barr ML Marboe C Rose EA Surgical management of cardiac pheochromocytoma: resection versus transplantation Ann Surg 1995 221 415 419 10.1097/00000658-199504000-00013 7726678 PMC1234592

[B30] Brown ML Zayas GE Abel MD Young WF Schaff HV Mediastinal paragangliomas: the mayo clinic experience Ann Thorac Surg 2008 86 946 951 10.1016/j.athoracsur.2008.04.105 18721588

[B31] Pachter MR Mediastinal nonchromaffin paraganglioma J Thorac Cardiovasc Surg 1963 45 152 160 13940959

[B32] Rufini V Treglia G Castaldi P Perotti G Calcagni ML Corsello SM Comparison of 123-I-MIBG SPECT-CT and 18 F- DOPA PET-CT in the evaluation of patients with known or suspected recurrent paraganglioma Nucl Med Commun 2011 32 575 582 10.1097/MNM.0b013e328345a340 21471850

